# When bronchial obstruction is not only asthma: a case of congenital cystic adenomatoid malformation type 0

**DOI:** 10.1186/2045-7022-3-S1-P24

**Published:** 2013-05-03

**Authors:** Luciana Indinnimeo, Valeria Lollobrigida, Taulant Melengu, Maria Palma Carbone, Azzurra Cesoni Marcelli, Francesca Occasi, Valentina De Vittori, Marzia Duse

**Affiliations:** 1 Università La Sapienza, Policlinico Umberto 1, Italy; 2 Università La Sapienza, Policlinico Umberto 1, Servizio di Allergologia ed Immunologia Pediatrica, Italy

## 

Congenital cystic adenomatoid malformation (CCAM) is one of the most common congenital lung anomalies. It is a rare pulmonary alteration, characterized by lung tissue dysplastic or hamartomatous, mixed with normal tissue. The injury is likely related to an insult during embryological development with altered terminal bronchiolar structures. We describe an unusual case of a boy came to our observation at the age of 12 years, for mild persistent bronchial asthma with exercise induced asthma, allergic to dust mites, pollens of grasses, Cypress and to epithelium of the cat. At birth, respiratory distress treated with oxygen therapy, which resolved on the second day of life. For the first two years of life, he had recurrent episodes of wheezing requiring bronchodilator therapy and oral corticosteroids; subsequently, he presented rare episodes of bronchospasm, and asthma induced from intensive exercise. He practiced martial arts at a competitive level. The routine spirometry showed FEV1 58.5% and MEF50 24.7%, values that were not reversible after salbutamol, despite therapy with CSI + LABA. The chest X-ray showed thickening of the right parietal pleura and of the apical one and evidence of fibrotic striae. The chest HRCT revealed mosaic bilateral parenchymal destruction, bronchiectasis and multiple formations nodules like, poly-lobed with blurred margins, the larger of a diameter of 12 mm. The bronchioloalveolar lavage was normal. Inflammatory, autoimmune, infectious and other congenital diseases were excluded. The histology of the lung biopsy obtained by thoracotomy surgery, suggested the diagnosis of CCAM type 0 without malignancy.

